# Short and long-term safety of MP29-02*: a new therapy for the treatment of allergic rhinitis

**DOI:** 10.1186/2045-7022-3-S2-O15

**Published:** 2013-07-16

**Authors:** David Price, Jean Bousquet, Peter Hellings, Glenis Scadding, Wytske Fokkens, Ullrich Munzel, Claus Bachert

**Affiliations:** 1 University of Aberdeen, Aberdeen, UK; 2 Hopital Arnaud de Villeneuve University Hospital, Montpellier, France; 3 University Hospitals Leuven, Dept of Otorhinolaryngology, Head and Neck Surgery, Leuven, Belgium; 4 The Royal National Throat, Nose and Ear Hospital, London, UK; 5 Academic Medical Center, Dept of Otorhinolaryngology, Amsterdam, the Netherlands; 6 Meda Pharma, Biostatistics & Market Access, Bad Homburg, Germany; 7 Ghent University Hospital, Dept of Oto-rhinolaryngology, Ghent, Belgium

## Background

MP29-02*, a novel intranasal formulation of azelastine hydrochloride (AZE) and fluticasone propionate (FP) provides significantly superior symptom relief to current first line therapy in patients with seasonal allergic rhinitis (SAR) and with chronic rhinitis [1,2].

## Objective

To evaluate the short- and long-term safety of MP29-02*.

## Method

4022 patients (>=12 years old) were randomized into 4, 14-day double-blind, placebo-controlled SAR trials to receive MP29-02*, AZE, FP or placebo nasal sprays (all given as 1 spray/nostril bid). 612 patients (>=12 years old) were randomized into a 1-year, open-label, active-controlled, parallel-group chronic rhinitis trial to receive MP29-02* (1 spray/nostril bid) or FP nasal spray (2 sprays/nostril qd). For all studies the total daily dose of AZE and FP was 548 g and 200 µg respectively. Safety was assessed by incidence, type, and severity of adverse events, vital signs and nasal examination.

## Results

In all SAR studies, the treatment-related adverse events (TRAEs) observed were those usually reported with AZE (dysgeusia) and FP (headache and epistaxis), did not exceed placebo in many instances (Table 1 shows results from a representative SAR study) and were 'mild' in the vast majority of cases. In the long-term study there was no evidence for an accumulation of TRAEs over time, any occurrence of late AEs and none were considered severe. < 3% of subjects discontinued from the study due to an AE. A SAE was reported by 3 MP29-02 subjects and 1 FP subject, but none were considered treatment-related. For all studies, changes in vital signs and nasal examinations were similar in all groups.

**Table 1:** 

MP4002 SAR study (14 days)				
	MP29-02* (n=207)	FP (n=207)	AZE (n=208)	Placebo (n=210)
TRAE n (%)	17 (8.2%)	14 (6.8%)	16 (7.7%)	8 (3.8%)
Dysgeusia	5 (2.4%)	2 (1.0%)	7 (3.4%)	1 (0.5%)
Epistaxis	2 (1.0%)	5 (2.4%)	4 (1.9%)	2 (1.0%)
Headache	1 (0.5%)	5 (2.4%)	1 (0.5%)	3 (1.4%)

Chronic rhinitis study (52 weeks)				
	MP29-02* (n=404)	FP (n=207)		

TRAE n (%)	38 (9.4%)	23 (11.1%)		
Dysgeusia	10 (2.5%)	1 (0.5%)		
Epistaxis	5 (1.2%)	1 (0.5%)		
Headache	4 (1.0%)	9 (4.3%)		

## Conclusion

MP29-02* was well tolerated following 14 day's use in SAR patients with a similar safety profile as standard therapies and placebo. MP29-02* is also safe for long-term use.

*Dymista
